# Alterations of the expression of *TET2* and DNA 5-hmC predict poor prognosis in Myelodysplastic Neoplasms

**DOI:** 10.1186/s12885-023-11449-2

**Published:** 2023-10-26

**Authors:** Ashikh A. Seethy, Karthikeyan Pethusamy, Tushar Kushwaha, Gaurav Kumar, Joyeeta Talukdar, Rekha Chaubey, Udayakumar Dharmalingam Sundaram, Manoranjan Mahapatra, Renu Saxena, Ruby Dhar, Krishna K. Inampudi, Subhradip Karmakar

**Affiliations:** 1 Department of Biochemistry, All India Institute of Medical Sciences, New Delhi, India; 2 Department of Biochemistry, All India Institute of Medical Sciences, Guwahati, India; 3 Department of Biophysics, All India Institute of Medical Sciences, New Delhi, India; 4 Department of Hematology, All India Institute of Medical Sciences, New Delhi, India; 5 Department of Hematopathology, Medanta – The Medicity, Gurgaon, India

**Keywords:** Myelodysplastic neoplasms, Myelodysplastic syndrome, AML-myelodysplasia related, Acute myeloid leukemia with myelodysplasia related changes, Epigenetics, TET2 protein, DNA sequence analysis, Molecular dynamics simulation, MDS in India

## Abstract

**Background:**

Myelodysplastic Neoplasms (MDS) are clonal stem cell disorders characterized by ineffective hematopoiesis and progression to acute myeloid leukemia, myelodysplasia-related (AML-MR). A major mechanism of pathogenesis of MDS is the aberration of the epigenetic landscape of the hematopoietic stem cells and/or progenitor cells, especially DNA cytosine methylation, and demethylation. Data on TET2, the predominant DNA demethylator of the hematopoietic system, is limited, particularly in the MDS patients from India, whose biology may differ since these patients present at a relatively younger age. We studied the expression and the variants of TET2 in Indian MDS and AML-MR patients and their effects on 5-hydroxymethyl cytosine (5-hmC, a product of TET2 catalysis) and on the prognosis of MDS patients.

**Results:**

Of the 42 MDS patients, cytogenetics was available for 31 sub-categorized according to the Revised International Prognostic Scoring System (IPSS-R). Their age resembled that of the previous studies from India. Bone marrow nucleated cells (BMNCs) were also obtained from 13 patients with AML-MR, 26 patients with de-novo AML, and 11 subjects with morphologically normal bone marrow. The patients had a significantly lower TET2 expression which was more pronounced in AML-MR and the IPSS-R higher-risk MDS categories. The 5-hmC levels in higher-risk MDS and AML-MR correlated with TET2 expression, suggesting a possible mechanistic role in the loss of TET2 expression. The findings on TET2 and 5-hmC were also confirmed at the tissue level using immunohistochemistry. Pathogenic variants of TET2 were found in 7 of 24 patient samples (29%), spanning across the IPSS-R prognostic categories. One of the variants – H1778R – was found to affect local and global TET2 structure when studied using structural predictions and molecular dynamics simulations. Thus, it is plausible that some pathogenic variants in TET2 can compromise the structure of TET2 and hence in the formation of 5-hmC.

**Conclusions:**

IPSS-R higher-risk MDS categories and AML-MR showed a reduction in TET2 expression, which was not apparent in lower-risk MDS. DNA 5-hmC levels followed a similar pattern. Overall, a decreased TET2 expression and a low DNA 5-hmC level are predictors of advanced disease and adverse outcome in MDS in the population studied, i.e., MDS patients from India.

**Supplementary Information:**

The online version contains supplementary material available at 10.1186/s12885-023-11449-2.

## Background

Myelodysplastic Neoplasms (MDS) is a heterogeneous group of clonal hematopoietic disorders, characterized by abnormal bone marrow morphology and bone marrow failure leading to peripheral cytopenia(s), and an increased risk of progression to acute myeloid leukemia (AML) [1–3]. AML developing in the context of prior MDS is referred to as AML, myelodysplasia related (AML-MR) [3]; 20–30% of individuals with MDS progress to AML-MR annually – thus, MDS is a pre-malignant condition [4]. Being a clonal disorder, the primary abnormality in MDS lies in the hematopoietic stem cells and/or progenitor cells (HSCPs), resulting in abnormal maturation and differentiation of these cells [5]. Epigenetic changes play a key role among the molecular alterations that instigate the pathogenesis of MDS – the driver mutations in MDS can lead to aberrations in chromatin modification, abnormalities in cohesin complex, and dysregulation of DNA methylation and de-methylation [2]. The latter is mediated by genes involved in methylation/de-methylation at the 5th position of cytosine in the DNA resulting in the formation or removal of 5-methyl cytosine (5-mC), respectively, and pathogenic variants in these genes are found in nearly 40% to 50% of MDS patients [6].

In the myeloid hematopoietic system, the primary enzyme that catalyzes the formation of 5-mC is DNA methyl transferase 3A (DNMT3A), while the predominant DNA 5-mC de-methylator is an Fe(II) and 2-keto glutarate dependent dioxygenase known as TET2 [7–9]. TET2 causes iterative oxidations of 5-mC, the products of which are acted upon by cellular DNA repair systems to restore cytosine in the erstwhile 5-mC locus [10]. The most stable, and hence, the most abundant product of TET2-mediated oxidation is 5-hydroxymethyl cytosine (5-hmC). This results in the negation of various biological effects brought about by 5-mC and 5-mC binding proteins – i.e., nucleosome remodeling, chromatin compaction, facilitation of higher order chromatin organization, and transcriptional repression [11]. TET2 is involved in the self-renewal of HSCs, lineage commitment, and terminal differentiation of hematopoietic cells into specific lineages [12].* TET2* nucleotide variants abrogating TET2 enzymatic activity, and hence a reduction in the 5-hmC levels in the bone marrow, are associated with various hematological neoplasms including AML [13, 14].

*TET2* pathogenic variants have been found in > 20% of MDS patients across multiple studies and they might play a role in the development of MDS, at least partially independent of other genetic risk factors [15–18]. The expression of *TET2* is also considerably reduced in the bone marrow nucleated cells (BMNCs), more so in the high-risk MDS groups [19, 20]. However, the effect of *TET2* nucleotide variants and that of reduced *TET2* expression on the expected reduction in 5-hmC levels is not conclusive, and reports on the effect of a reduced 5-hmC level, if any, on the prognosis of MDS are conflicting [21, 22]. To ascertain their probable clinical significance, we checked for the presence of *TET2* pathogenic variants, *TET2* gene expression levels, and the 5-hmC levels in MDS and AML-MR patients from India. We also performed in silico analysis using structure prediction and molecular dynamics simulation to study the effect of one of the *TET2* pathogenic variants identified. MDS in India is rather unique due to its varied age of presentation [21], and the current study is the first of its kind to assess DNA demethylation in this peculiar patient cohort.

## Methods

### Selection of study subjects and sample collection

The study subjects included patients with a confirmed diagnosis of primary myelodysplastic neoplasms (as per WHO 2022 classification of MDS) [1] who had not received any disease-modifying treatment, and patients with de novo AML, or AML-MR. The control arm of the study included patients who had diagnosis of non-malignant conditions and a morphologically normal bone marrow (e.g., patients with peripheral blood cytopenias who were on a trial of vitamin B_12_ due to suspected deficiency where the marrow was found to be morphologically normal at the time of bone marrow sampling, and patients with non-malignant causes of hypersplenism who presented with cytopenias but had a morphologically normal marrow). Only adult patients (≥ 18 years of age at the time of sample collection) were included. Those patients with therapy related MDS or AML, MDS/myeloproliferative neoplasm (MPN) overlap syndromes, chronic myelomonocytic leukemia (CMML), and acute promyelocytic leukemia were excluded. The study was performed in accordance with the relevant guidelines and regulations (Declaration of Helsinki) and was approved by the Institute Ethics Committee for Post Graduate Research, All India Institute of Medical Sciences, New Delhi, vide Letter No. IECPG-309/07.09.2017 dated September 14, 2017. Written informed consent was obtained from all the study subjects from whom any biological sample was collected. Up to 2.5 mL of bone marrow aspirate was collected from the study subjects in EDTA vial for obtaining bone marrow nucleated cells for DNA and RNA isolation. 4 μm sections that were cut from formalin fixed paraffin embedded bone-marrow biopsy specimens onto poly-L-lysine were also collected. The other details like clinico-hematological parameters and cytogenetics were obtained from the patients’ medical records and hospital information system.

### Isolation of BMNCs, DNA, and RNA

A protocol optimised for downstream extraction of DNA and RNA was adopted while isolating BMNCs [22]. Briefly, the bone marrow aspirate was transferred to a 15 mL centrifuge tube and was centrifuged at 4 °C. After the removal of the supernatant, an equal volume of 1X RBC lysis buffer (BioLegend, San Diego, CA) was added to the tube, followed by gentle mixing and incubation at room temperature for 10 min. The tube was centrifuged, the supernatant was removed, and the same was repeated after the addition of 1 mL 1X RBC lysis buffer, this time in a 1.5 mL microcentrifuge tube. Following high-speed centrifugation, the pellet was washed with 1 mL phosphate buffered saline (PBS). The pellet was then suspended in Buffer RLT Plus (Qiagen, USA) (with β-mercapto-ethanol added). The cells in the buffer were homogenized by passing through a 20-gauge needle at least 5 times. The homogenized cells in the Buffer RLT Plus were stored at—80 °C for subsequent DNA and RNA isolation using AllPrep DNA/RNA Mini Kit (Qiagen, USA), which enabled the isolation of DNA and RNA from the same starting material in one go. The RNA isolation involved in-column DNase digestion to remove any contaminant DNA. The extracted DNA and RNA were quantified using a nano-spectrophotometer. A_260/280_ and A_260/230_ values of ≥ 1.8 and ≥ 2 were considered suggestive of good-quality DNA and RNA, respectively. Aliquots of the isolated DNA and RNA were also subjected to agarose gel electrophoresis to check for the integrity of the nucleic acids and detection of contamination with RNA or DNA, as the case may be. Only those DNA and RNA samples that met adequate quality standards and had sufficient quantity were subjected to further analysis.

The input amount of DNA for the 5-hmC assay (described later) was 100 ng in a volume of 4 μL – i.e., 25 ng/μL. Since the input DNA amount was critical due to the sensitive nature of the assay, the DNA concentration in the samples used for the assay was estimated using a dye-based method (QuantiFluor® ONE dsDNA System – Promega Corporation – Madison, WI), where a fluorescent double-stranded DNA-binding dye (504 nm Ex/ 531 nm Em) specific only for double-stranded DNA was used. The fluorescence after dye-binding was estimated by Quantus™ Fluorometer (Promega Corporation – Madison, WI).

### cDNA synthesis and quantitative real time PCR

1 μg of the extracted RNA was used for cDNA synthesis with random hexamer priming using Verso cDNA synthesis kit (Thermo Scientific, EU) according to the manufacturer's protocol. 1 μL of the cDNA (equivalent to 50 ng input RNA) was used for the subsequent qPCR reactions. The primers used for the qPCR reactions are listed in Supplementary Table 1. All the primers were designed to span an exon-exon junction in order to nullify the inadvertent amplification of genomic DNA targets by these primers. The cDNA was amplified using DyNAmo Flash SYBR Green qPCR Kit (Thermo Scientific, EU). The reactions were performed in triplicates, with negative and -RT (without reverse transcriptase) controls, and the runs were validated by performing a melt-curve analysis. The AriaMx Real-Time PCR System (Agilent Technologies.Inc) was used for performing the runs. The fold-change for the Gene of Interest (GOI) was calculated in the test samples in comparison to the control samples using the using the ΔΔCt method, using Glyceraldehyde 3-phosphate dehydrogenase (*GAPDH*) as the reference gene [23]. The latter was selected from a panel of reference genes as it showed the most consistent expression in the hematopoietic cell lines and marrow aspirate samples. The results were log-transformed and were expressed as log_2_-fold change with respect to the controls.

### Quantitative Assay for 5-hmC

Colorimetric assay based on a one-step ELISA for quantification of global DNA hydroxymethylation was performed using MethylFlash Global DNA Hydroxymethylation (5-hmC) ELISA Easy Kit (Colorimetric) (Epigentek, USA) using manufacturer’s protocol. The input amount of DNA for the assay was 100 ng (in a volume of 4 μL – i.e., 25 ng/μL). All the samples, standards, and the negative control were assayed in duplicates and the average absorbance of the negative control was subtracted from the samples and the standards. The standard curve was generated by plotting the absorbance of the different positive control samples on the Y-axis against the known 5-hmC percentage of these samples in the X-axis. A second order polynomial curve was graphed, and the second order polynomial regression equation in the form Y = aX^2^ + bX + c was obtained, were X = 5-hmC%, Y = absorbance, a and b are slope 1 and slope 2, respectively. The percentage 5-hmC in the test samples were calculated by the formula

$$5-\mathrm{hmC\% }=\frac{{\left({\mathrm{b}}^{2}+4\mathrm{aY}\right)}^{0.5}-\mathrm{b}}{2\mathrm{a}}\mathrm{x}\frac{100\mathrm{\%}}{\mathrm{S}}$$

where S is the input DNA amount (100 ng in the current study)

The absorbance in test samples was compared with that of standards to obtain percentage 5-hmC levels in the test samples.

### Immunohistochemistry (IHC) for TET2 and 5-hmC

Formalin fixed paraffin embedded (FFPE) bone marrow biopsy tissue sections were deparaffinized by heat and multiple xylene washes, followed by removal of xylene by graded washes with ethanol and rehydration in de-ionized water. The sections were further dipped in 10 mM citrate buffer (pH: 6) and were heated for 40 min for antigen unmasking, followed by three washes with Tris wash-buffer. The sections were placed for peroxide block for 10 min and the Tris wash was repeated. The non-specific binding of antibodies was blocked with Protein Block followed by three washes with phosphate buffered saline (PBS). The sections were incubated with the primary antibody (TET2 – mouse monoclonal antibody – C15200179, stock: 1 μg/μL, Diagenode – USA in 1:200 dilution, or 5-hmC – rat monoclonal antibody—C15220001-50, stock: 1 μg/μL, Diagenode – USA in 1:250 dilution) for 90 min in the dark, at room temperature, followed by two washes with Tris buffer, and then with biotinylated secondary antibodies followed by washing. After incubation with streptavidin-peroxidase complex, and washing, freshly prepared di-amino benzidine (DAB) (along with peroxidase substrate) was applied on the slides. The slides were immersed in distilled water as soon as a crisp brown nuclear staining was seen on monitoring with a microscope. The slides were counterstained with hematoxylin, dehydrated in graded alcohol, were then passed through xylene, and were mounted with Dibutylphthalate Polystyrene Xylene (DPX). Nuclear positivity was assessed in mononuclear cells and the percentage nuclear positivity was calculated in the stained cells. The cell counts were repeated thrice for determining the percentage of positive cells within the cellular areas of the marrow.

### Sequencing of *TET2* gene

Since *TET2* is a relatively large gene with exons 3–11 of the gene coding for 2002 amino acids, individually amplifying each of these exons and performing individual Sanger sequencing was technically cumbersome. Hence, exome sequencing (NGS) with an average depth of 100X was adopted to test for *TET2* variants. For this 50 ng of DNA from each sample was used for Whole Exome Sequencing (WES) library preparation using Twist Library Preparation Kit (Twist Biosciences) followed by enrichment of the exome using ‘Twist Fast Hybridization Target Enrichment Protocol’ which was done in 3 pooled samples. Paired end Illumina sequencing using Illumina Hiseq 4000 NGS platform was carried out to generate 2 × 150 bp reads at an average sequencing depth of 100X. The general protocol for data analysis was obtained from Scaria V, et al. [24], where the fastq files from the sequencer were checked for quality using FASTQC, followed by trimming of the adapters and the low quality reads using Trimmomatic-0.36. An average Phred score of 30 and a Phred score of 20 in a sliding window of 5 were maintained. Stampy with Burrows-Wheeler-Alignment was used for mapping of the reads to the reference genome hg38. The aligned reads were sorted using SAMtools and the alignment quality was verified using Qualimap. Reads aligning to multiple loci were removed using Picard, and variant calling was performed with Platypus. BCFTools was used to screen the vcf files for the depth of sequencing (minimum depth of 20). The reads were visualized with Integrated Genome Viewer. Annotation of the variants was performed using ANNOVAR for genomic co-ordinates, chromosome location, population databases, in silico predictions, and known disease databases. The variants were first filtered by selecting those having splicing altering potential based on dbscSNV-ADA score and RF-score > 0.6 [25] and those located in the exons. Exonic synonymous variants were excluded, and frequency filter was applied with a cut-off of minor-allele frequency < 10% using gnomAD-Exome, gnomAD-Genome, ExAc and 1000 genome project databases. In-silico pathogenicity assessment of the mis-sense variants and indels was performed by SIFT (‘Deleterious’ and ‘Unknown’), Polyphen2-HDIV (‘Probably Damaging’, ‘Damaging’, and ‘Unknown’), and a CADD-Phred Score ≥ 15 (with at least 2 of these databases giving a concordant result). Frame-shift variants leading to premature termination codons were also considered pathogenic. The datasets [aligned bam files of the WES reads to the TET2 locus] generated and analysed during the current study are available in the NCBI-SRA repository [Accession no. SRP441583].

### Sanger sequencing for validation of exome data

To validate some of the variants in *TET2* observed after exome sequencing, the region of genomic DNA around the observed variant was amplified by conventional PCR using Phusion® High-Fidelity DNA Polymerase – New England Biolabs (for 30 cycles to minimize PCR artefacts). The presence of the amplicon was confirmed by agarose gel electrophoresis of a small aliquot of the completed PCR reactions. The amplicon was then subjected to a PCR purification using SMARTPURE PCR clean-up kit (Eurogentec, Belgium). Purified amplicon was quantified using QuantiFluor® ONE dsDNA System (Promega Corporation – Madison, WI) and 40 ng of the amplicon was used for Sanger Sequencing along with other reaction components using ABI 3730XL (Thermo Scientific, USA) for capillary electrophoresis and data analysis. The forward and reverse chromatograms obtained from the sequencing software was examined to assess the quality of the reactions. The sequences obtained were aligned to the Ensembl human gene sequence using EMBOSS Water nucleotide alignment, for confirmation of the variants. The chromatograms were visualized for confirming the nature and the approximate frequency of the variant.

### Structure prediction of TET2

The TET2 protein domain architecture is shown in Fig. 4a for clarity. The 3D structure of the TET2 catalytic domain (TET2-CD) was predicted in order to model the Low Complexity Insert (LCI), which is missing in the crystal structure (PDBID:5D9Y) formed by residues 1481–1844 [26]. The structure of the LCI was modeled using RoseTTAFold on the Robetta Server, [27] followed by modeling of TET2 from 1127–1938 residues along with DNA using PDBID:5D9Y as a template. The model was validated using the Ramachandran plot and other metrics provided by the MolProbablity server [28]. The modeled structure was further refined through molecular dynamics simulations. The deleterious mutation, H1778R, was modeled using Pymol.

### Molecular dynamics simulations

The modeled structure of TET2 and its variant forms were subjected to molecular dynamics simulations to understand the influence of the H1778R mutation on its local and global structure. Simulations were carried out using the GROMACS software suite (version 2021.5) with AMBER99SB-ILDN force field (Abraham, SoftwareX 2015). The proteins and protein-DNA complexes were solvated in a dodecahedron box with a TIP3P water model followed by neutralization with an appropriate number of sodium or chloride ions. The neutralized system was energy minimized using the steepest descent algorithm followed by equilibration under NVT [constant number (N), constant-volume (V), and constant-temperature (T)] and NPT [as for NVT, but pressure (P) is regulated] ensemble sequentially. Production simulation was run using a leapfrog dynamic integrator with a step size of 2 fs for a timescale of 200 ns, considering periodic boundary conditions in all three dimensions. Post simulation analysis was done after eliminating periodic boundary conditions using modules available in GROMAC and in-house python scripts. Simulations were carried out for wild type and H1778R variant TET2 in both apo state and DNA bound form.

### Statistical analysis

The statistical analysis was performed using GraphPad Prism 8.0. The data were expressed in median and inter-quartile range or mean ± SD. Testing for normality was done by Kolmogorov–Smirnov test. Most of the data in the current study were non-parametric. These data were compared across different groups using Mann–Whitney U-test or Kruskal–Wallis test (when number of groups were > 2), since the effect of outliers on such analyses is minimal [29]. Quantitative variables were compared between each other using Spearman’s rank correlation coefficient. A *p*-value of < 0.05 was considered statistically significant.

## Results

### MDS and AML in India affect a relatively younger population

In the current study, samples were collected from a total of 42 patients with MDS, 13 patients with AML-MR, 26 patients with de-novo AML, and 11 subjects with morphologically normal bone marrow. Cytogenetics data was available for 31 of the 42 MDS patients, and this, along with other laboratory parameters like hemoglobin, absolute neutrophil count, platelet count and bone marrow blast percentage, was used to obtain the Revised International Prognostic Scoring System (IPSS-R) score [20]. The MDS patients were also subtyped based on marrow morphology and cytogenetics as per the 2022 WHO Classification [1]. The baseline demographics of the study subjects from this study are given in Table 1. The age of the study subjects was comparable (Supplementary Figure 1a), and the median age was ≤ 50 years across all the groups. This is in contradiction to the data from the US and elsewhere where nearly 86% of MDS patients belonged to the age-group of 60 years or more, where the median age at diagnosis was found to be 76 years [30]; similarly, the median age at diagnosis for AML in the US was 65 years and 60% of AML patients were aged ≥ 60 years [31]. However, lower age at presentation in this study (Supplementary Figure 1b) was similar to that reported in the previous studies from our center [21, 32–34] and elsewhere in India on MDS [35, 36] and AML, [37] though the reason for such a varied observation is yet to be elucidated. The age distribution across the IPSS-R subcategories as well as the WHO subtypes of MDS remained nearly uniform (Supplementary Figure 1c and d).

**Table 1:** Demographics of the study subjects

Characteristics	Number
Total number of subjects	MDS: 42
	AML-MR: 13
	De novo AML: 26
	Controls: 11
Median Age in years (minimum and maximum in parenthesis)	MDS: 49 (18 to 90 years)
	AML-MR: 50 (18 to 65 years)
	De novo AML: 36 (18 to 69 years)
	Controls: 41 (24 to 74 years)
Sex (M:F)	MDS: 1.625:1
	Male: 26
	Female: 16
	AML-MR: 1.17:1
	Male: 7
	Female: 6
	De novo AML: 0.625:1
	Male: 10
	Female: 16
	Controls: 1.2:1
	Male: 6
	Female: 5
Cytogenetics of MDS patients (*n* = 42)	46,XX or 46,XY: 17
	del(5q): 3
	–7 or del(7q): 3
	Complex (≥ 3 abnormalities): 3
	del(20): 1
	t(3;5): 1
	Others: 3
	Not available: 11
IPSS-R risk category of MDS patients (*n* = 42)	Very low: Nil
	Low: 10
	Intermediate: 12
	High: 3
	Very high: 6
	Not available: 11
Subtype of MDS (*n* = 42)	MDS-LB: 15
	MDS-LB-RS: 4
	MDS-5q: 3
	MDS-IB: 12
	MDS-h: 8

### Low expression of *TET2* was seen in AML-MR and higher risk IPSS-R MDS patients

**Fig. 1:**
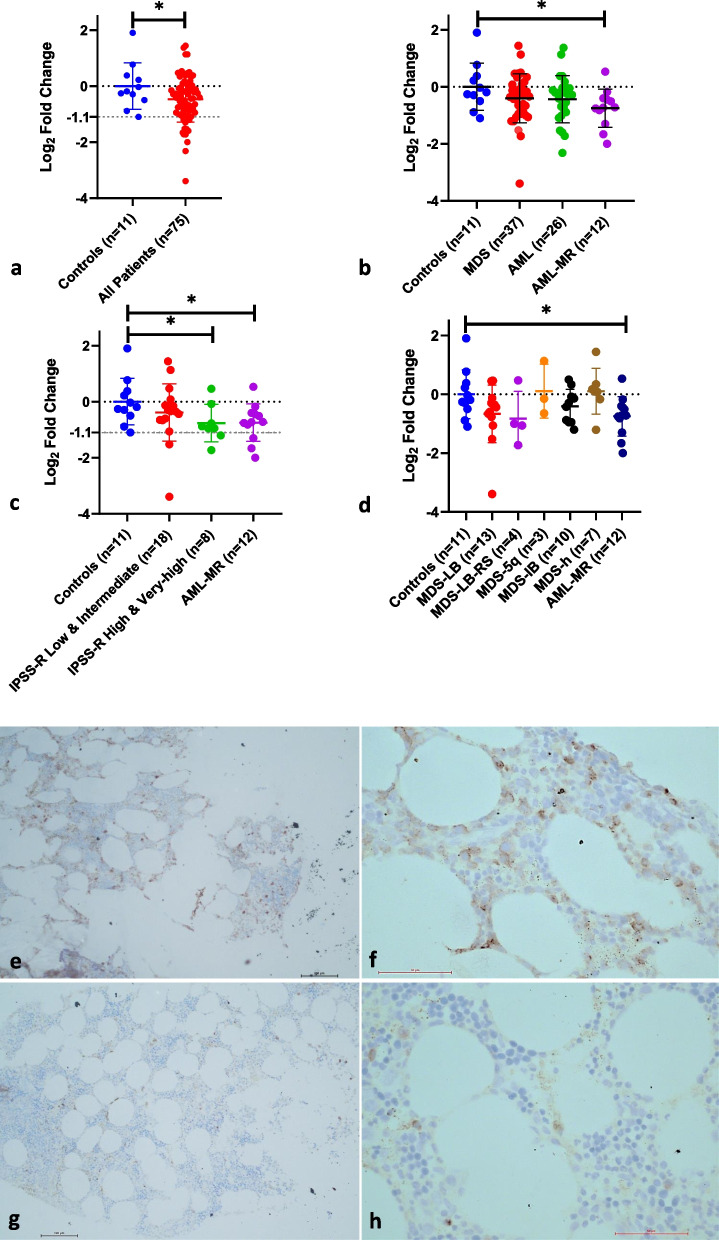
*TET2* expression in the study subjects: **a**-**d**: Using qPCR: **a**. All the study subjects. **b**. Individual patient groups. **c**. IPSS-R sub-categories of MDS. **d**. WHO subtypes of MDS. All the statistical comparisons were made using the Mann–Whitney U test, and * denotes a *p*-value < 0.05. **e**–**h**: Immunohistochemistry for TET2 in the bone marrow biopsy FFPE sections of two of the study subjects. The left panels (**e** and **g**) show magnification at 10X objective, and the right panels (**f** and **h**) show the magnification at 40X objective. TET2-positive cells show a brown staining. The biopsy sample in the top panel (**e** and **f**) had 30–35% nuclear positivity for TET2 compared to the one in the lower panel (**g** and **h**), which had < 5% nuclear positivity for TET2. The sample in the top panel had ~ 5 times more expression of *TET2* in qPCR relative to the one in the lower panel

On quantification of the mRNA expression using qPCR, the patients in general showed a significantly lower expression of *TET2* when compared to controls (log2-fold change: -0.5, *p*-value: < 0.05; Mann–Whitney U test) (Fig. 1a). The expression below a cut-off log_2_-fold change of -1.1 was observed only among the patients. As for the individual groups, the expression was significantly lower in AML-MR compared to controls (log2-fold change: -0.8, *p*-value: 0.03; Mann–Whitney U test) (Fig. 1b); the expression was lower but not statistically significant in MDS and AML, compared to controls. Further, when the expression was analyzed across different IPSS-R risk categories, it was found to be significantly lower in the higher IPSS-R risk categories (*p*-value: < 0.05; Mann–Whitney U test) and this reduction in the expression was similar to that in AML-MR (Fig. 1c). Though certain MDS sub-types like MDS-LB, MDS-LB-RS, and MDS-IB showed a decrease in *TET2* expression with respect to controls and other subtypes, these were not statistically significant (Fig. 1d). The expression of *TET2* obtained in the qPCR was further validated by performing immunohistochemistry for TET2 protein in the FFPE sections of bone marrow biopsy from two of the subjects studied with high and low *TET2* expression in qPCR, and the results of the IHC corroborated with the expression values obtained in qPCR (Fig. 1e–h).

### A decreased *TET2* expression is associated with a reduction of global DNA 5-hmC levels

**Fig. 2:**
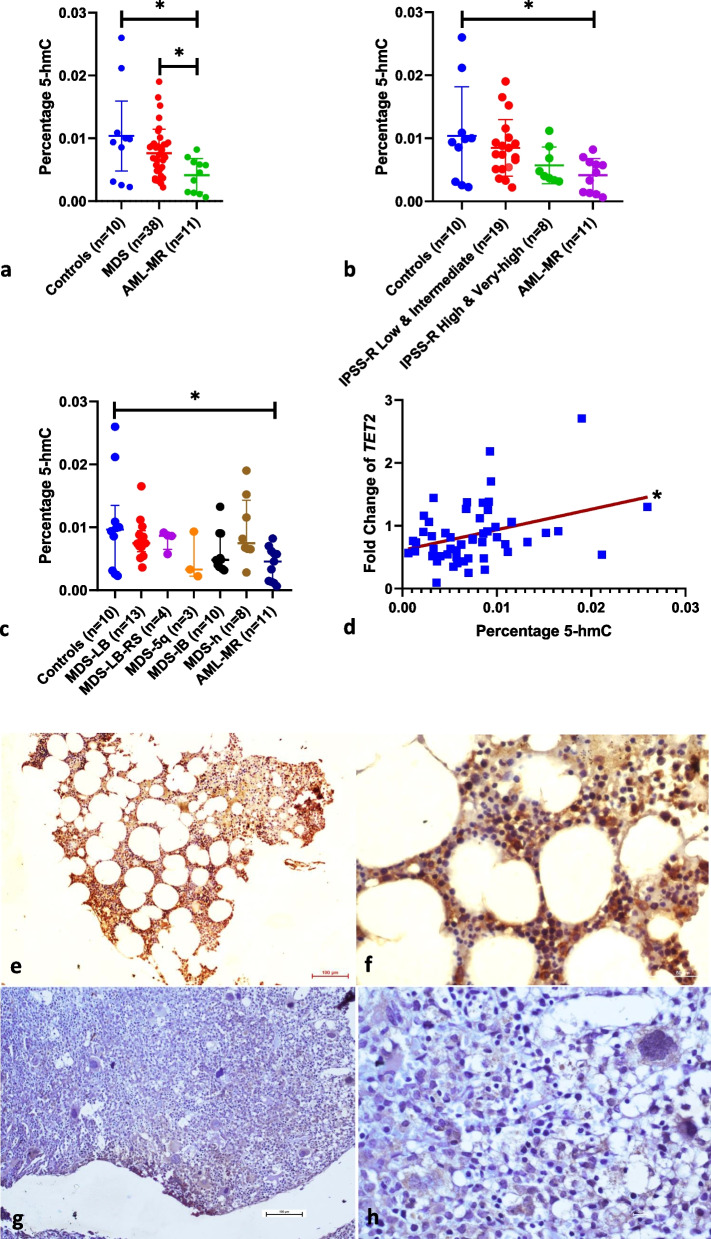
DNA 5-hmC levels in the study subjects: **a**-**d**: Percentage 5-hmC levels **a**. Individual patient groups. **b**. IPSS-R sub-categories of MDS. **c**. WHO subtypes of MDS. **d**. Correlation of 5-hmC Levels with *TET2* expression. The statistical comparisons in a-c were made using the Mann–Whitney U test, and in d were made using Spearman correlation; * denotes a *p*-value < 0.05. **e**–**h**: Immunohistochemistry for 5-hmC in the study subjects' bone marrow biopsy FFPE sections. The left panels (**e** and **g**) show magnification at 10X objective and the right panels (**f** and **h**) show magnification at 40X objective. 5-hmC positive cells show brown staining. The samples are arranged with control in the top panel (**e** and **f**), which showed 20 to 25% nuclear positivity for 5-hmC, and AML-MR (**g** and **h**) in the lower panel, which has < 5% nuclear positivity. A total of 23 samples were subjected to IHC for 5-hmC – the images of the rest of the samples are provided in Supplementary Figure 2 and in ‘ Supplementary file—All IHC Images’

Since the first stable product from TET2 mediated catalysis is 5-hmC, we quantified the levels of 5-hmC in the DNA of the study subjects (except de novo AML) using a colorimetry-based immuno-assay. The median percentage 5-hmC levels in AML-MR (4.6 × 10^–3^) differed significantly from that of controls (9.6 × 10^–3^) and MDS (7.2 × 10^–3^) (*p*-value: 0.02 and < 0.05 respectively, Mann–Whitney U test) (Fig. 2a). The levels were lower, but not statistically significant, in the IPSS-R higher risk categories compared to controls and IPSS-R lower risk categories (Fig. 2b). Also, there was no significant difference in the 5-hmC levels across different MDS subtypes (Fig. 2c). The pattern of reduction in the 5-hmC levels in the higher risk IPSS-R categories and AML-MR was similar to that of the *TET2* expression shown in Fig. 1. The mRNA expression of *TET2* showed a positive significant positive correlation with the percentage 5-hmC levels in the DNA (*p*-value: 0.03; Spearman correlation) (Fig. 2d). The reduction in 5-hmC levels in AML-MR and higher risk categories of MDS was also confirmed by the examination of FFPE tissue sections from bone marrow biopsy using immunohistochemistry for 5-hmC. IHC was carried out for 23 samples (AML-MR:3, IPSS-R Very-high risk MDS: 2, IPSS-R High risk MDS: 2, IPSS-R Intermediate risk MDS: 6, IPSS-R Low risk MDS: 4, and Controls: 5). Control samples and lower IPSS-R risk categories showed higher expression of 5-hmC while the higher risk categories and AML-MR samples had much lower 5-hmC expression (Fig. 2e-h and Supplementary Figure 2).

### Pathogenic *TET2* variants were found in nearly 30% of the patients

**Fig. 3:**
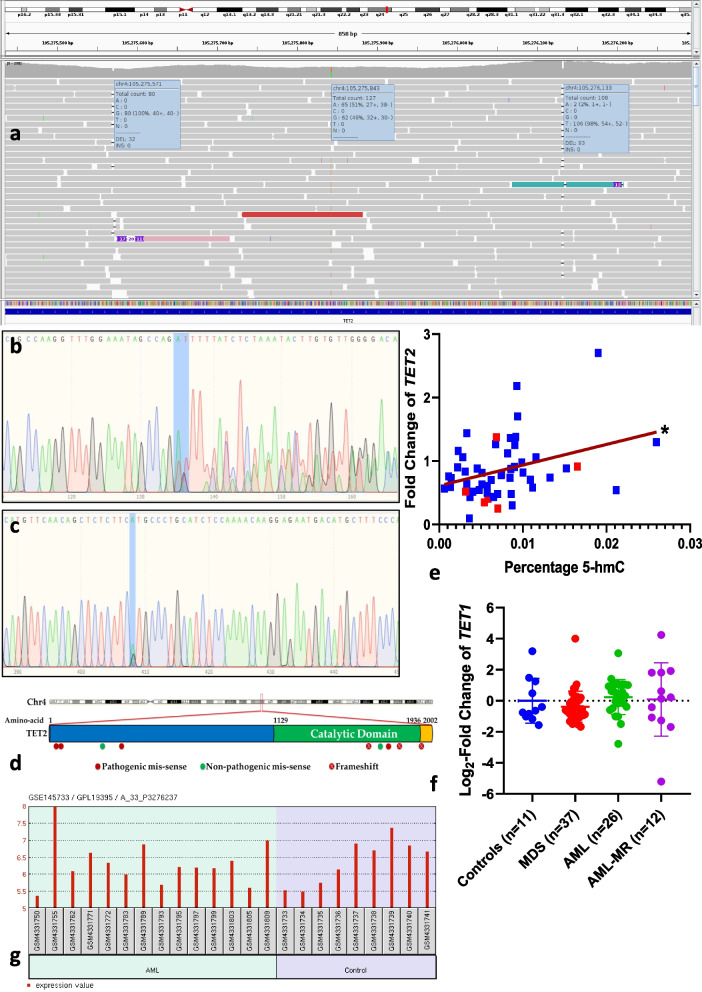
Sequencing of *TET2* gene: **a**. *TET2* variants (c.5060_5061del:p.Q1687fsX3, c.A5333G:p.H1778R, c.5622_5623del:p.E1874fsX2) from one of the AML-MR samples visualized in Integrative Genomics Viewer. **b**. Visualization of Exon11:c.5060_5061del:p.Q1687fsX3 using Sanger sequencing. The alteration in the bases following the frameshift deletion at the region highlighted is also visualized. **c**. Sanger sequencing showing *TET2* Exon11:c.A5333G:p.H1778R in Sample I1. **d**. The location of the variants with respect to the catalytic domain of TET2. **e**. Correlation between fold-change of *TET2* and percentage DNA 5-hmC levels. The samples with pathogenic variants in *TET2* are shown in red. **f**. *TET1* expression in the study subjects did not show any significant difference across the groups (Kruskal Wallis test and Mann–Whitney U test) **g**. Analysis of GSE145733 using NCBI GEO – GEO2R. A comparison of AML-MR with controls did not show any significant difference in *TET3* expression (Spot id for *TET3*: A_33_P3276237)

DNA from BMNC samples of 24 patients (17 MDS and 7 AML-MR) were assessed for the presence of pathogenic variants of *TET2* using exome sequencing, where 7 of the 24 samples (5 MDS and 2 AML-MR) tested positive (Supplementary Table 2). Of these, one patient with AML-MR had multiple aberrations in the *TET2* gene including 2 frameshift deletions (Fig. 3a); the only other frameshift variant was found in a patient with high-risk MDS (Supplementary Table 2). To validate the results of the exome sequencing, the presence of two of the variants was further confirmed using Sanger sequencing (Fig. 3b-c). A number of these variants were localised to the catalytic domain of TET2 (Fig. 3d). Next the effect of the pathogenic variants on the levels of 5-hmC was checked for. Most of the samples with a pathogenic variant in *TET2* showed a lower level of 5-hmC; however, this could not be considered causal, since majority of these samples also had a lower level of *TET2* mRNA expression (Fig. 3e). Further, since some of the samples also showed a lower 5-hmC levels despite a high *TET2* mRNA expression even in the absence of *TET2* mutations, the expression of *TET1* which is a paralog of *TET2* [38] was studied in the study subjects using quantitative real time PCR, although *TET1* is not considered as a major DNA hydroxy-methylating enzyme in the hematopoietic system. The expression of *TET1* however did not vary significantly across the patient groups (Fig. 3f) or IPSS-R categories, giving rise to speculations about other mechanisms that can alter the 5-hmC levels in the BMNCs, a likely candidate being the 3^rd^ paralog of TET proteins – *TET3*. Yet, analysis of publicly available AML-MR and MDS datasets (GSE5881 and GSE145733) from NCBI-GEO using GEO2R did not show any significant alteration of *TET3* expression in AML-MR and MDS when compared to healthy controls (Fig. 3g).

### *TET2* p.H1778R variant affects the local and global structure of TET2

**Fig. 4:**
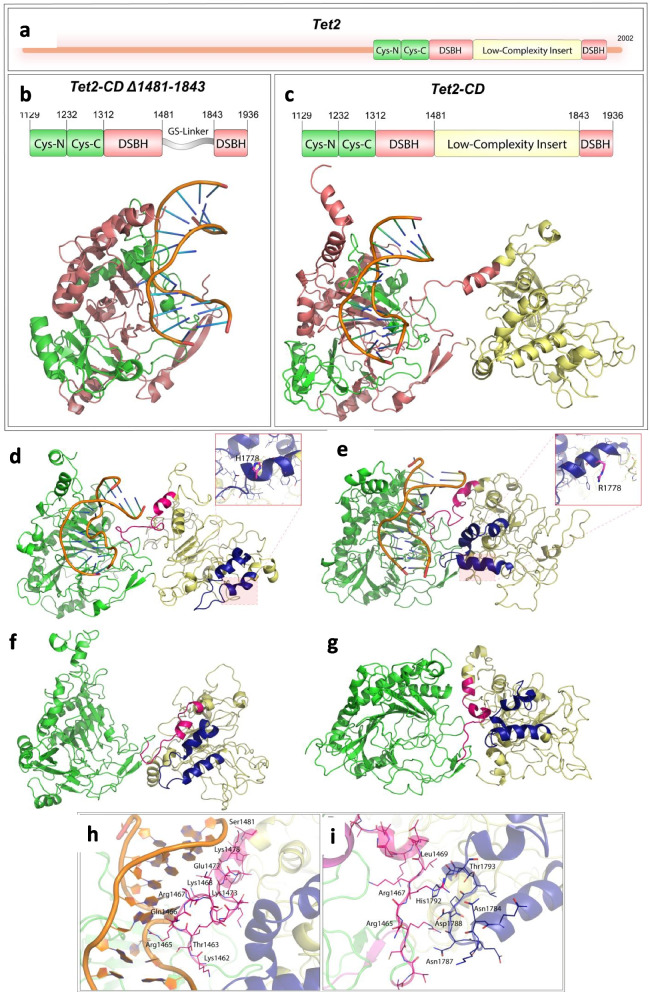
Structure of TET2 and the effect of H1778R variant: **a**. Gene structure of full-length TET2. **b**. Gene Structure of TET2-CD Δ1481-1843 and the crystal structure of TET2-CD (PDBID:5D9Y). The crystal structure includes coordinates for the residues 1132–1481 and 1842–1929 along with the DNA; the low complexity insert has been replaced with a 15-residue Glycine-Serine linker. **c**. The gene structure of TET2-CD and the modelled structure include the low complexity insert shown in yellow. The Cys-rich domain is shown in green and the DSBH domain in red. **d**. The structure of TET2-CD in complex with DNA was observed after 200 ns simulation for the wild type. **e**. H1778R variant (**f**) without DNA of TET2-CD and (**g**) without DNA of H1778R variant TET2-CD. The low-complexity insert is shown in yellow, with the N-terminal region (residues 1462–1481) highlighted in pink, and the helix-turn-helix (formed by the residues 1772–1807) is shown in navy blue. (**h**) Interactions between the DNA and LCI region in the TET2-CD H1778R variant and (**i**) TET2-CD H1778R variant LCI intradomain interactions in the absence of DNA, which were absent in the wildtype TET2-CD

A molecular dynamics simulation was performed to study the effect of one of the pathogenic amino-acid variants in TET2 – p.H1778R – on the protein structure and stability. The TET2 protein domain architecture is shown in Fig. 4a. The crystal structure of the minimally active TET2 protein (residues 1128–1936 Δ1481-1843) includes a cysteine-rich domain followed by the double-stranded β-helix (DSBH) domain which is formed by a core of double-stranded beta helix structure also known as a jelly roll motif. DNA binds to the L1 and L2 loops of the Cys-Rich domain above the DSBH domain (Fig. 4b). The pathogenic variant H1778R lies in the Low Complexity Insert (LCI) within the DSBH domain of the *TET2* gene. To assess the impact of H1778R on the structure of TET2, we modelled the structure of the catalytic domain (CD) of TET2 along with the low complexity insert since the existing crystal structures of TET2 are devoid of the LCI region (DOI for the model: https://www.modelarchive.org/doi/10.5452/ma-9k1ka). The structure of the LCI (residues 1481–1846) was modelled using RosettaFold on the Robetta server. The predicted structure shows a globular protein having an α/β fold with a significant unstructured region between the well-formed secondary structures (Fig. 4c). The structure of the TET2-CD, in complex with DNA, was then modelled using comparative modelling on the Robetta Server. The modelled structure shows a distinct exterior domain for the LCI in concordance with the previously reported predictions based on multiple sequence alignment [39]. Further, the catalytic domain of TET2-CD with H1778R variant was also built through comparative modelling using Pymol.

**Fig. 5:**
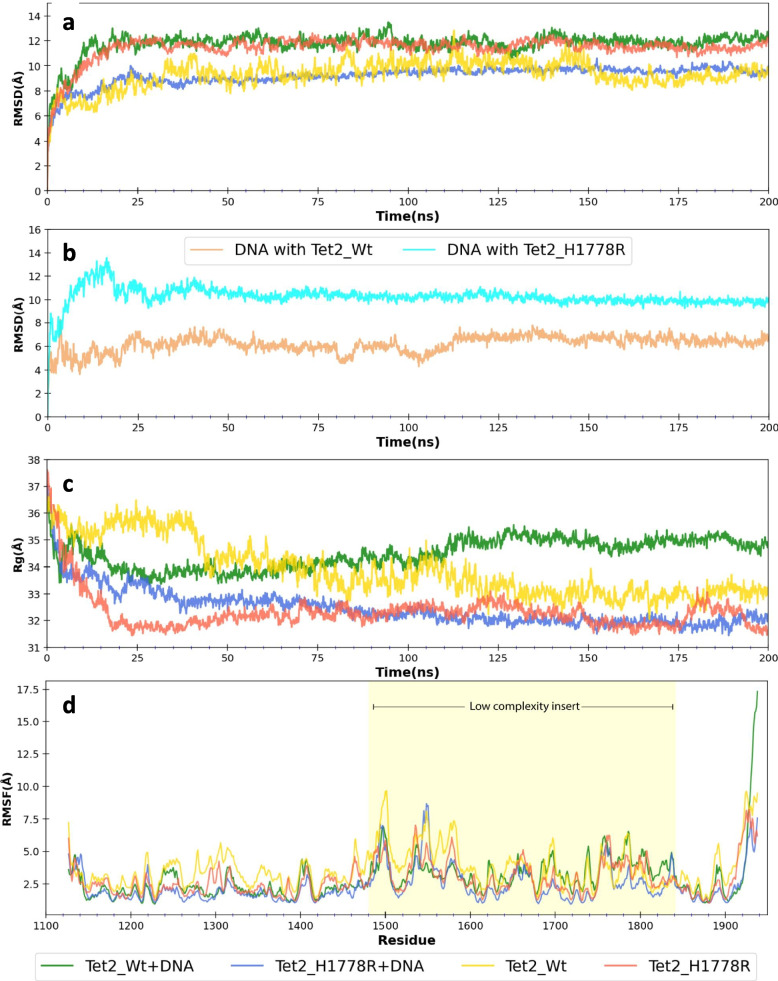
Molecular dynamics simulations: a. Backbone RMSD. b. DNA RMSD. c. Rg and (d) Root Mean Squared Fluctuation (RMSF) were observed during the 200 ns simulations for the different forms of TET2-CD

Molecular dynamics simulations were performed to understand the structural influence of the H1778R variant on the catalytic domain of TET2-CD. We subjected the modelled structures of TET2-CD harboring the LCI region, in the native and variant forms, for 200 ns simulations in the presence of DNA and absence of DNA. The TET2-CD proteins and protein-DNA complexes were stable throughout the 200 ns simulations but displayed significant differences in their global dynamics, as shown in Fig. 4d to g. The LCI region demonstrated significantly more rotational and translational movement compared to the DSBH domain. The variant, H1778R, seemed to alter the global and local structure of the TET2-CD, as shown in Fig. 4e and g. More residue fluctuations were observed in the DNA-binding region of TET2-CD in the absence of DNA. The LCI region was more compact in the variant owing to increased intramolecular interactions (Fig. 4e). The TET2-CD_H1778R was more stable and compact, compared to the wild-type TET2-CD, as evident from the Root Mean Squared Deviation (RMSD) and Radius of gyration (Rg), respectively, as shown in Fig. 5a-d. In the case of the variant, the DNA was surrounded by the Cys-rich domain and the LCI domain, while in the wild type, interactions of LCI with DNA were diminished (Fig. 4d).

The N-terminal region of the LCI (residues 1462–1481), harbouring positively charged residues, showed interactions with DNA in both wild-type and variant TET2-CD. While in the wild type, these interactions were limited to the DNA backbone, the variant formed extensive base-specific interactions along with the DNA backbone (Fig. 4h). Also, the helix-turn-helix formed by the residues 1772 to 1807 showed interactions with the DNA backbone in the case of variant TET2-CD. This helix-turn-helix was very far from the DNA in the case of wild-type TET2-CD. In the absence of DNA, the positively charged N-terminus of LCI formed interactions with this helix-turn-helix (Fig. 4i). Also, interactions between the Cys-rich domain and LCI were observed in the variant TET2-CD, which were absent in the wild-type TET2-CD. This interface was formed by the N-terminus region of LCI and the residues R1253, K1254, Y1255, P1278, R1279, and D1314 of the Cys-C subdomain. The H1778 residue is part of the helix formed by residues 1768–1780. During the simulation, the hydrophobic H1778 in the wild type limited solvent exposure by forming intramolecular interactions, unlike the R1778 in the variant, which was predominantly solvent exposed.

In our patient samples, though two of the samples harboured the TET2 p.H1778R variant, one of them had co-occurring variants in *TET2* including frameshifts, and the effect of the p.H1778R was hence not exclusive (Supplementary Table 2). The alternate sample, from a patient with IPSS-R intermediate risk and a normal bone marrow cytogenetics, showed the pathogenic variant in 58% of the reads (Supplementary Figure 3a). This patient showed a relatively low 5-hmC levels compared to the rest of the patient cohort, which is partly explained by the low mRNA expression levels of *TET2* (Supplementary Figure 3b-c). This patient also was found to have a low TET2 protein expression and 5-hmC immunostaining on IHC (Supplementary Figure 3d-e), corroborating with the possibility of altered catalytic activity of TET2, as elucidated by molecular dynamics simulation.

## Discussion

The role of *TET2* in the pathogenesis of MDS and its progression to AML-MR was investigated in this study, with emphasis on the gene expression of *TET2*, the presence or absence of pathogenic variants in *TET2*, and the potential effects of these two on the levels of 5-hmC in the DNA of the patients. The cohorts used were MDS, AML, and AML-MR patients from India, whose demographics varied from the extensively studied Western population, especially in the context of age, and the proportion of patients with favourable cytogenetics and outcome. To the best of our knowledge, this is the first study on the role of *TET2* in the pathogenesis of MDS in this population, and the aforementioned peculiarities of Indian MDS patients were replicated here also. A pertinent question at this juncture is the validity of prognosticating MDS patients in such a population as ours using the IPSS-R scoring system which presumes that the median age of the patients is 70 years. Whether incorporation of age (using age-adjusted IPSS-R or IPSS-RA, or other tools) into IPSS-R could have a clinical implication while making treatment decisions needs to be addressed by studies designed for the same, especially when it has already been observed that younger Indian MDS patients progress rapidly to AML-MR than their elderly counterparts [21]. MDS patients with IPSS-R ‘very-low’ risk was absent in this study among the patients recruited – this could be because certain cytogenetic features like -Y and del(11q) that are associated with a good prognosis [20] were not found in the patients studied; an alternate explanation is the possibility of underdiagnosis and late diagnosis of MDS patients in India.

The expression of *TET2* was significantly lower in the patients when compared to controls, with the low expression more pronounced in the higher risk categories (very-high- and high-risk categories combined in IPSS-R prognostic system) of MDS and in AML-MR. A low expression of *TET2* has been reported to be associated with an adverse prognosis in MDS, [17] with *TET2* expression inversely correlating with IPSS prognostic scores [40]. The blurring of the boundary between higher-risk MDS and AML-MR observed in context of *TET2* expression has been reiterated in the 2022 WHO classification of MDS (previously termed as ‘myelodysplastic syndromes’ and now renamed to ‘myelodysplastic neoplasms’) which mentions ‘any blast-based cut-off (for distinguishing MDS and AML) is arbitrary and cannot reflect the biologic continuity naturally inherent in myeloid pathogenic mechanisms’ [41]. In the present study, the expression of *TET2* in de novo AML was low compared to the controls, but the difference observed was not statistically significant. This could be due to various reasons – foremost, the alterations in the methylation of DNA have been seen prominent in AML-MR and MDS, rather than in de novo AML and normal CD34^+^ cells [42]; further, the expression of *TET2* progressively decreases with the increase in severity of de novo AML, [43] but such a risk-stratification was not performed in the de novo AML patients in the current study.

Biological effects of TET2 stem from two major independent processes – the catalytic activity on 5-mC and the interaction of TET2 with other proteins [38]. The pattern of 5-hmC levels in this study closely followed that of the *TET2* expression, and the positive correlation between these two parameters corroborated this finding; a similar finding was also observed in other studies on *TET2* expression and 5-hmC levels [44]. The percentage 5-hmC levels in the study subjects were similar to other studies on 5-hmC levels in the hematopoietic system, further validating the current results [45]. The effects of protein interactions involving TET2 in MDS and AML-MR were not addressed in the present study.

The frequency of the pathogenic variants of *TET2* found in this study was similar to that observed across multiple studies on the same [15–18]. Since this is the first study of the mutational profile of *TET2* in the Indian population in any disease, similar data from the study population is lacking for any further comparison. Since the DNA from only the BMNCs was sequenced in the current study, the nature of the variants – whether somatic or germline – cannot be concluded. Many of the variants had an allele frequency of ~ 50% in the reads obtained (Fig. 3a), but this could very well be due to the larger mutational burden arising as a result of the clonal nature of the condition. Instances of germline variants in *TET2* leading to familial malignancies are extremely rare, but such reports are available in patients with lymphomas and myeloid malignancies, but not particularly in MDS [46, 47]. In our study, none of the patients had a history of familial segregation of the disease; hence the likelihood of germline variants is minimal. Most of our samples with a pathogenic variant in *TET2* also had a concurrent low expression of *TET2*, thus precluding any solid conclusions on the effect of these mutations, including those in the catalytic domain of TET2, on the catalysis mediated by TET2. Since the pathogenic variants in *TET2* were found across the risk groups of MDS, it is also likely that these variants could have been acquired by the mutant clones early in the pathogenesis of MDS, though the more deleterious variants like those leading to frameshift alterations were confined to the higher-risk groups. Our results that low *TET2* expression, but not pathogenic variants in *TET2*, has an impact on prognosis is in accordance with the findings of a 2017 meta-analysis of 14 studies by Lin Y, et al. [48]. We also observed that a simultaneous reduction in the 5-hmC levels in samples with low *TET2* expression, which was earlier reported in a study in Chinese population [49]. The same has also been observed in cell line and animal studies [50, 51].

Since some of the samples showed a lower 5-hmC levels despite a high *TET2* mRNA expression even in the absence of *TET2* mutations, we also studied the expression of other paralogs of *TET2*— expression of *TET1* did not vary significantly across the patient groups studies, and analysis of publicly available AML-MR and MDS datasets did not show any significant alteration of *TET3* expression in AML-MR and MDS when compared to healthy controls. However, some of the recent studies are contradictory in this regard and these indeed assign a role to TET3 in MDS with low *TET2* expression, in compensating and restoring the 5-hmC levels [52].

In the current study, patients harboring the H1778R variant in *TET2* was found to have a low TET2 protein expression and 5-hmC immunostaining on IHC, and this variant was further subjected to molecular dynamics simulation studies. The less conserved LCI has been predicted to have regulatory roles in the TET gene family through interaction with DNA, [53] protein–protein interactions, or probable post-translational modifications [39, 54]. The crystal structure of minimally active TET2-CD harbors a Glycine-Serine linker (GS linker) in place of the LCI, proximal to the major groove of DNA. As observed in the modelling studies, the interactions of the LCI with DNA propound it as a possible regulatory mechanism. Although not described, the previous TET2 truncation studies suggest the LCI be a negative regulator of TET2 activity since the minimally active TET2 domain has higher activity than the full-length TET2 [55]. The LCI might exert its influence on the activity of TET2 through direct interactions of its N-terminus with DNA or the Cys-rich domain, as observed in the simulations (Fig. 4a). Enhanced interactions of LCI with DNA were observed in the case of variant TET2-CD with H1778R, and in the absence of DNA, the LCI formed interface interactions with the Cys-rich domain (Fig. 4). The variation H1778R was present on the crucial helix-turn-helix motif of the LCI and was found to affect its local and global structure. Since low TET2 activity was observed in patients harbouring the variant TET2 (H1778R), this pathogenic variant augments the possible inhibitory action of LCI on the catalytic activity of TET2.

A shortcoming of the current study is that BMNCs were used for nucleic acid isolation rather than CD34^+^ cells isolated from the samples. However, the use of BMNCs for gene expression and transcriptome studies is not an uncommon practice. Indeed, the widely used cancer data repositories like The Cancer Genome Atlas (TCGA) use data from the transcriptome of BMNCs as such, rather than CD34^+^ purified fractions, in their studies on AML [56, 57]. The use of BMNCs also has a translational advantage as such studies, when validated, can easily be adopted into clinical setting and diagnostics [58]. Our study also lacked cytogenetics data for nearly 1/4th of the patients, which prevented the categorization of these patients according to IPSS-R, and their inclusion in multiple comparisons carried out in this study. Finally, the minor allele frequency (MAF) of nucleotide variants including that in *TET2* are not available in the public domain for the Indian population, thrusting us to rely on databases like ExAC and gnomAD to screen for MAF cut-offs.

## Conclusions

IPSS-R higher risk MDS categories and AML-MR showed a reduction in *TET2* expression, which was not apparent in lower-risk MDS. The formation of 5-hmC, the first step in DNA 5-methyl cytosine demethylation, was impaired in higher-risk MDS and AML-MR, suggestive of a possible mechanistic role of low *TET2* expression and also of the continuum of higher-risk MDS and AML in context of loss of *TET2* function. Though it is plausible that pathogenic variants in *TET2* can lead to decreased *TET2* mediated catalysis and formation of 5-hmC, the current study cannot conclusively prove this potential association. Overall, a decreased *TET2* expression and a low DNA 5-hmC level are predictors of advanced disease and adverse outcome in MDS in the population studied, i.e., MDS patients from India.

### Supplementary Information

**Additional file 1:** **Supplementary Figure 1.** a. Age distribution of the study subjects was similar across the 2 groups (Mann-Whitney U-test) b. More than 75% of patients were younger than 60 years 3 when individual MDS patients were sorted in the increasing order of their age. c. Age 4 distribution across IPSS-R subcategories was similar (Kruskal-Wallis test). d. Age distribution 5 did not vary across MDS subtypes (Kruskal-Wallis test). **Supplementary Figure 2.** Immunohistochemistry for 5-hmC in the study subjects' bone 8 marrow biopsy FFPE sections. The left panels (a, c, e, and g) show magnification at 10X 9 objective, and the right panels (b, d, f, and h) at 40X objective. 5 –hmC positive cells show 10 brown staining. The samples are arranged IPSS-R low risk on top (a and b), followed by 11 intermediate risk (c and d), high risk (e and f), and very high risk (g and h). The percentage of 12 nuclear positivity for 5-hmC in these samples is 10%, 5-10%, < 5%, and < 5%, respectively. 13 Additional IHC images showing 5-hmC in the controls, IPSSR-Low, IPSSR-Intermediate, IPSSR-14 High and AML-MRC samples. **Supplementary Figure 3.** Deleterious effects of *TET2 *p.H1778R on catalytic activity: a. *TET2 *18 variant (c.A5333G:p.H1778R) from an IPSS-R intermediate risk patient visualized in 19 Integrative Genomics Viewer showing the frequency of the pathogenic variant across all the 20 reads b. The percentage of 5-hmC of the same patient is shown using a star. c. *TET2 *expression 21 in the same patient is shown using a star. d. Immunohistochemistry for 5-hmC in the patient's 22 bone marrow biopsy FFPE sections showed < 5% nuclear positivity. e. Immunohistochemistry 23 for TET2 in the patient's bone marrow biopsy FFPE sections showed 30-35% nuclear positivity. 24 The magnification in Figures d and e are at 40X objective. **Supplementary Table 1.** List of primers used in this study. **Supplementary Table 2.** Pathogenic variants of TET2 in patient samples.

## Data Availability

The datasets [aligned bam files of the WES reads to the TET2 locus] generated and analysed during the current study are available in the NCBI-SRA repository [Accession no. SRP441583]. The structure of the catalytic domain (CD) of TET2 along with the low complexity insert can be accessed from https://www.modelarchive.org/doi/10.5452/ma-9k1ka.

## Abbreviations

5-mC: 5-Methyl cytosine
5-hmC: 5-Hydroxymethyl cytosine
AML: Acute myeloid leukemia
AML-MR: AML, myelodysplasia related
BMNCs: Bone marrow nucleated cells
CD: Catalytic Domain
CMML: Chronic myelomonocytic leukemia
DAB: Di-amino-benzidine
DNMT: DNA methyl-transferases
DPX: Dibutylphthalate Polystyrene Xylene
DSBH: Double stranded beta helix
ELISA: Enzyme Linked Immuno-Sorbent Assay
FFPE: Formalin fixed paraffin embedded
GAPDH: Glyceraldehyde 3-phosphate dehydrogenase
GOI: Gene of interests
GS-linker: Glycine-Serine linker
HSC: Hematopoietic stem cell
HSCPs: Hematopoietic stem cells and/or progenitor cells
IHC: Immunohistochemistry
IPSS-R: Revised International Prognostic Scoring System
IPSS-RA: Age-adjusted IPSS-R
LCI: Low complexity insert
MAF: Minor allele frequency
MDS: Myelodysplastic neoplasms
MDS-5q: MDS with low blasts and isolated 5q deletion
MDS-h: MDS, hypoplastic
MDS-IB: MDS with increased blasts
MDS-LB: MDS with low blasts
MDS-LB-RS: MDS with low blasts and ring sideroblasts
NCBI-GEO: National Center for Biotechnology Information—Gene Expression Omnibus
NCBI-SRA: National Center for Biotechnology Information – Sequence Reads Archive
NGS: Next generation sequencing
PBS: Phosphate buffered saline
qPCR: Quantitative real-time polymerase chain reaction
Rg: Radius of gyration
RMSD: Root Mean Squared Deviation
RMSF: Root Mean Squared Fluctuation
SIFT: Sorting Intolerant From Tolerant
TCGA: The Cancer Genome Atlas
TET: Ten eleven translocation proteins
WES: Whole Exome Sequencing
WHO: World Health Organisation
