# Supplementary material for: Alterations of the expression of TET2 and DNA 5-hmC predict poor prognosis in Myelodysplastic Neoplasms
Source: BMC Cancer. 2023 Oct 26;23:1035. doi: 10.1186/s12885-023-11449-2 (PMC10601240; doi:10.1186/s12885-023-11449-2)
Supplement: Supplementary file 1 — Additional file 1: Supplementary Figure 1. a. Age distribution of the study subjects was similar across the 2 groups (Mann-Whitney U-test) b. More than 75% of patients were younger than 60 years 3 when individual MDS patients were sorted in the increasing order of their age. c. Age 4 distribution across IPSS-R subcategories was similar (Kruskal-Wallis test). d. Age distribution 5 did not vary across MDS subtypes (Kruskal-Wallis test). Supplementary Figure 2. Immunohistochemistry for 5-hmC in the study subjects' bone 8 marrow biopsy FFPE sections. The left panels (a, c, e, and g) show magnification at 10X 9 objective, and the right panels (b, d, f, and h) at 40X objective. 5 –hmC positive cells show 10 brown staining. The samples are arranged IPSS-R low risk on top (a and b), followed by 11 intermediate risk (c and d), high risk (e and f), and very high risk (g and h). The percentage of 12 nuclear positivity for 5-hmC in these samples is 10%, 5-10%, < 5%, and < 5%, respectively. 13 Additional IHC images showing 5-hmC in the controls, IPSSR-Low, IPSSR-Intermediate, IPSSR-14 High and AML-MRC samples. Supplementary Figure 3. Deleterious effects of TET2 p.H1778R on catalytic activity: a. TET2 18 variant (c.A5333G:p.H1778R) from an IPSS-R intermediate risk patient visualized in 19 Integrative Genomics Viewer showing the frequency of the pathogenic variant across all the 20 reads b. The percentage of 5-hmC of the same patient is shown using a star. c. TET2 expression 21 in the same patient is shown using a star. d. Immunohistochemistry for 5-hmC in the patient's 22 bone marrow biopsy FFPE sections showed < 5% nuclear positivity. e. Immunohistochemistry 23 for TET2 in the patient's bone marrow biopsy FFPE sections showed 30-35% nuclear positivity. 24 The magnification in Figures d and e are at 40X objective. Supplementary Table 1. List of primers used in this study. Supplementary Table 2. Pathogenic variants of TET2 in patient samples. [file 12885_2023_11449_MOESM1_ESM.pdf]

Figure Legends (Supplementary figures)

Supplementary Figure 1: a. Age distribution of the study subjects was similar across the groups (Mann-Whitney U-test) b. More than 75% of patients were younger than 60 years when individual MDS patients were sorted in the increasing order of their age. c. Age distribution across IPSS-R subcategories was similar (Kruskal-Wallis test). d. Age distribution did not vary across MDS subtypes (Kruskal-Wallis test)

Supplementary Figure 2: Immunohistochemistry for 5-hmC in the study subjects' bone marrow biopsy FFPE sections. The left panels (a, c, e, and g) show magnification at 10X objective, and the right panels (b, d, f, and h) at 40X objective. 5 –hmC positive cells show brown staining. The samples are arranged IPSS-R low risk on top (a and b), followed by intermediate risk (c and d), high risk (e and f), and very high risk (g and h). The percentage of nuclear positivity for 5-hmC in these samples is 10%, 5-10%, < 5%, and < 5%, respectively. Additional IHC images showing 5-hmC in the controls, IPSSR-Low, IPSSR-Intermediate, IPSSR-High and AML-MRC samples.

Supplementary Figure 3: Deleterious effects of *TET2* p.H1778R on catalytic activity: a. *TET2* variant (c.A5333G:p.H1778R) from an IPSS-R intermediate risk patient visualized in Integrative Genomics Viewer showing the frequency of the pathogenic variant across all the reads b. The percentage of 5-hmC of the same patient is shown using a star. c. *TET2* expression in the same patient is shown using a star. d. Immunohistochemistry for 5-hmC in the patient's bone marrow biopsy FFPE sections showed < 5% nuclear positivity. e. Immunohistochemistry for TET2 in the patient's bone marrow biopsy FFPE sections showed 30-35% nuclear positivity. The magnification in Figures d and e are at 40X objective.

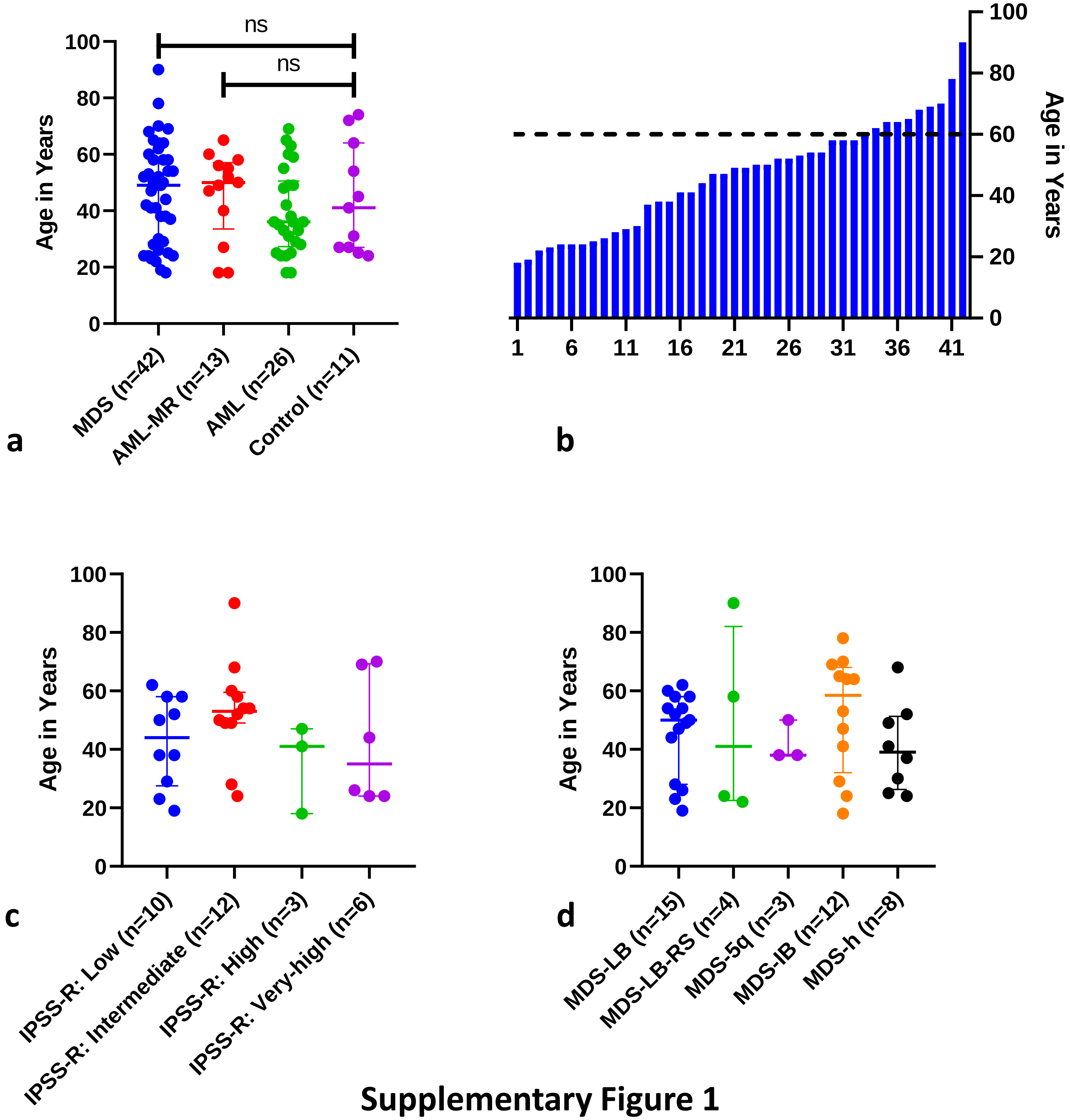

Supplementary Figure 1

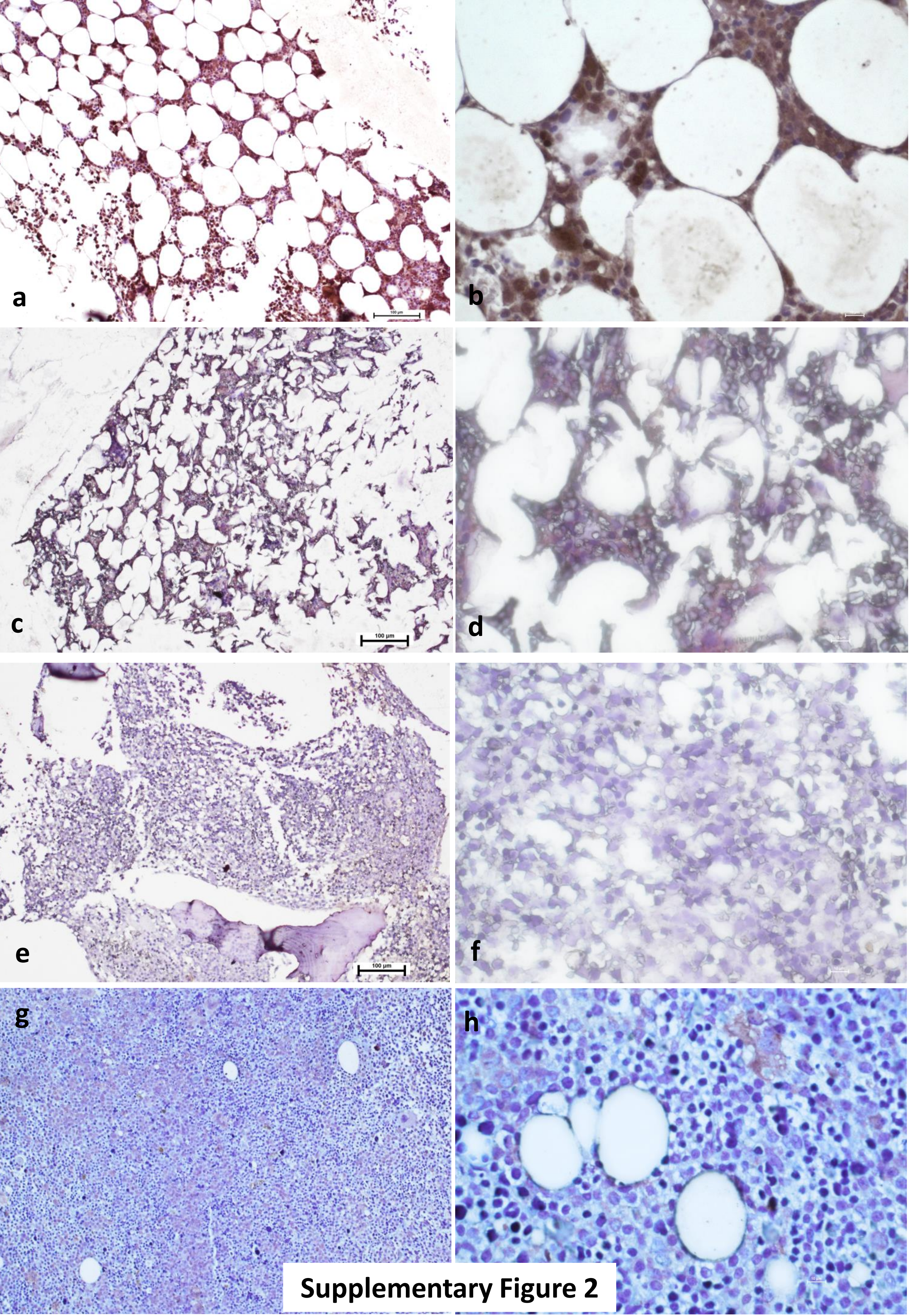

Supplementary Figure 2

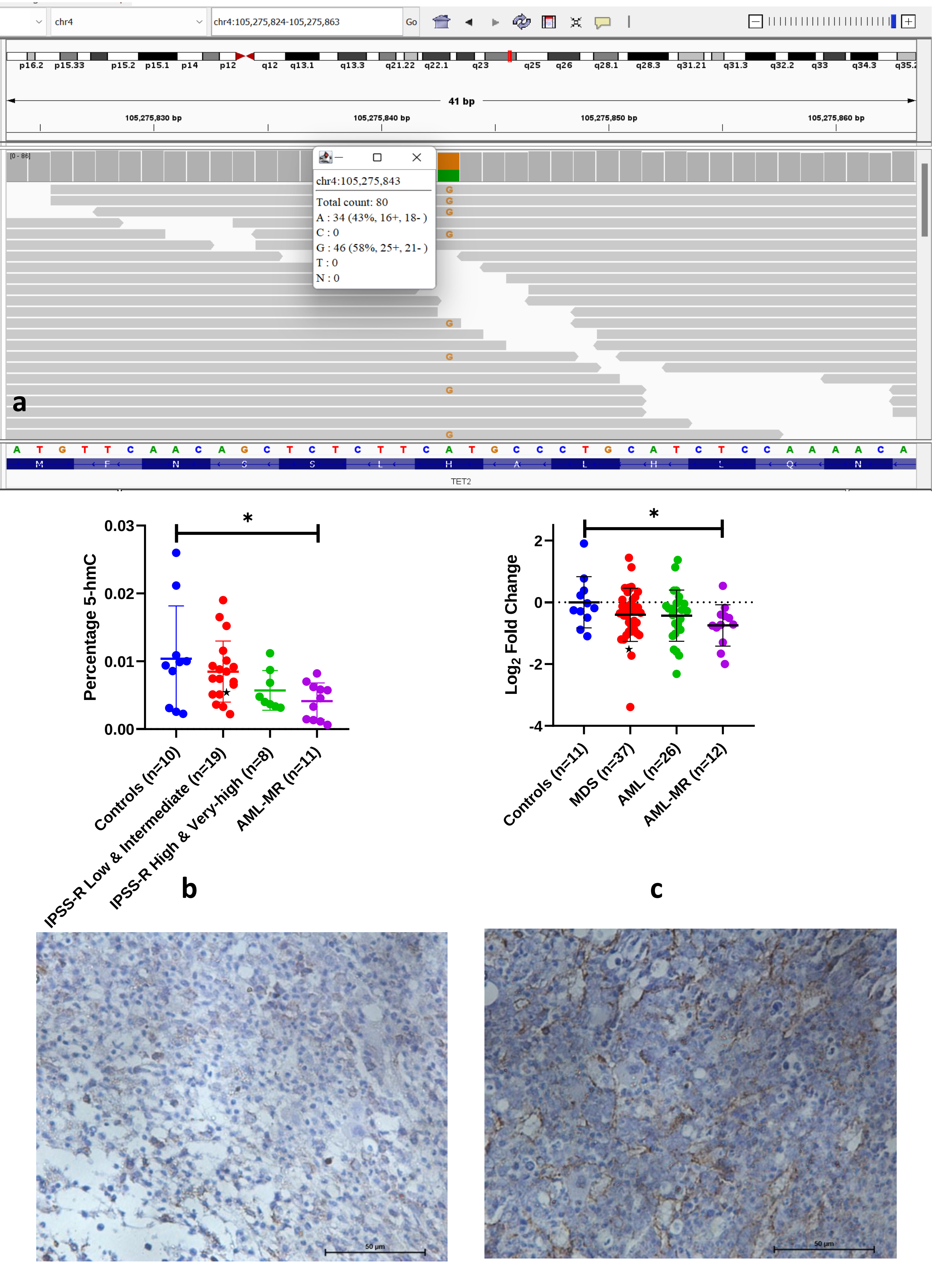

d

Supplementary Figure 3

e

Control

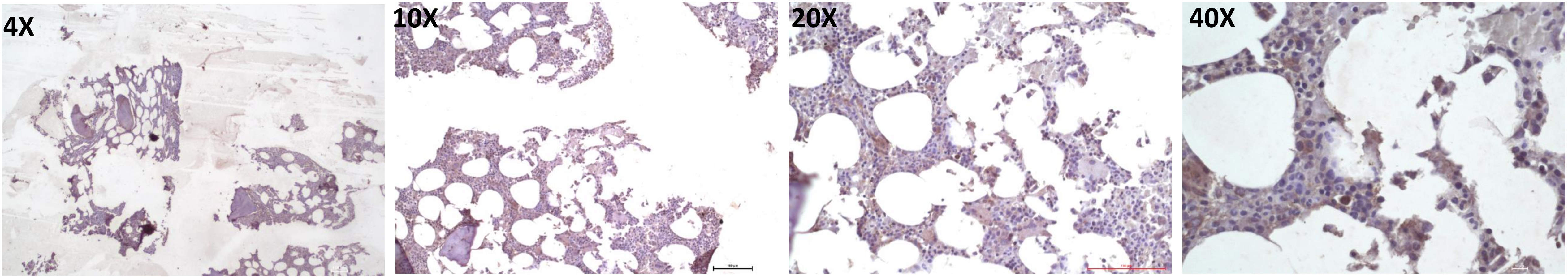

Control

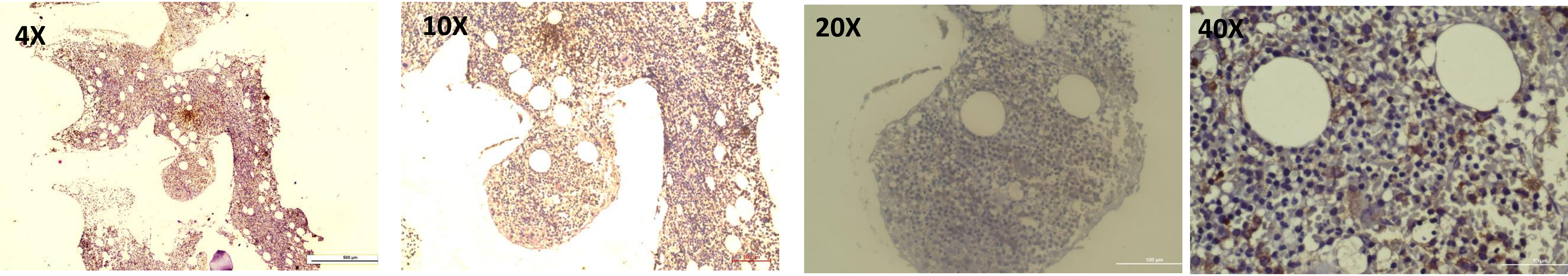

Control

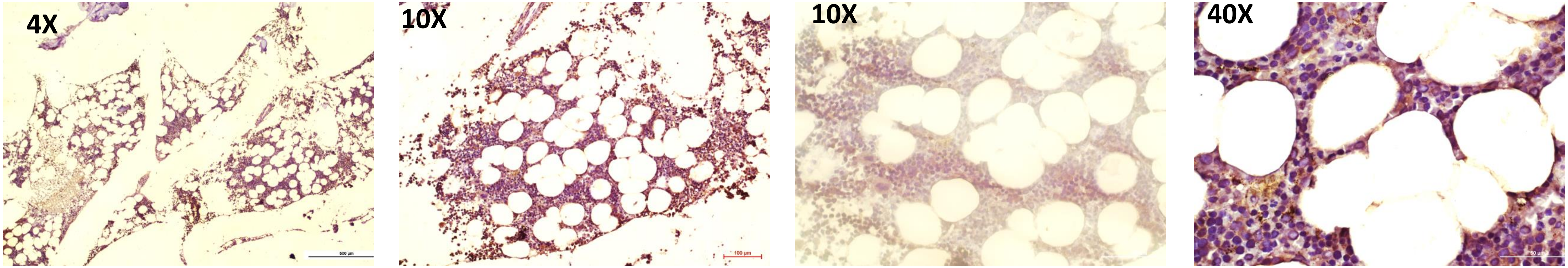

Control

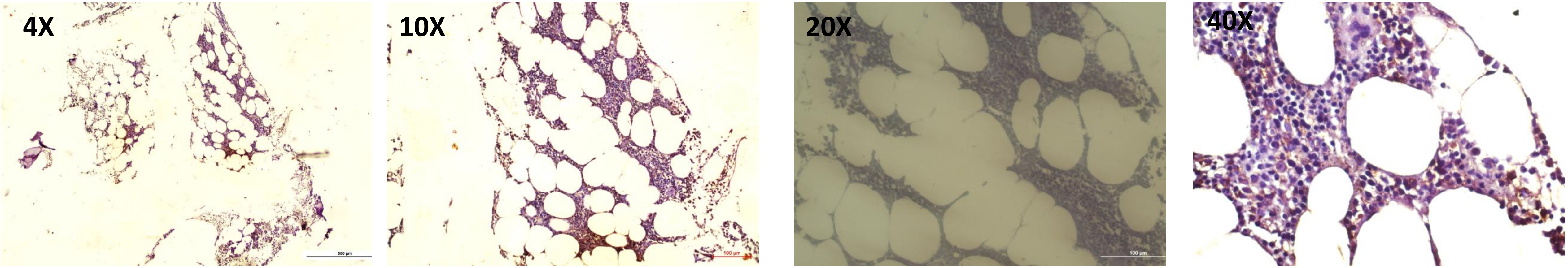

Control

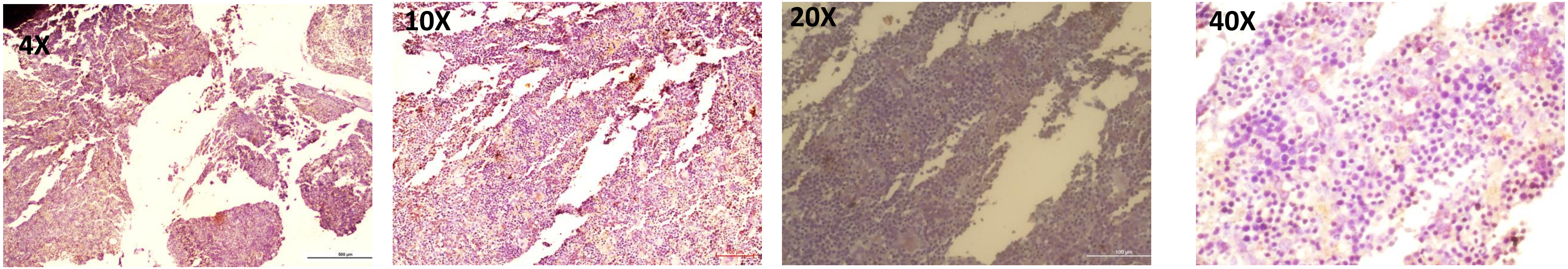

IPSS-R Low

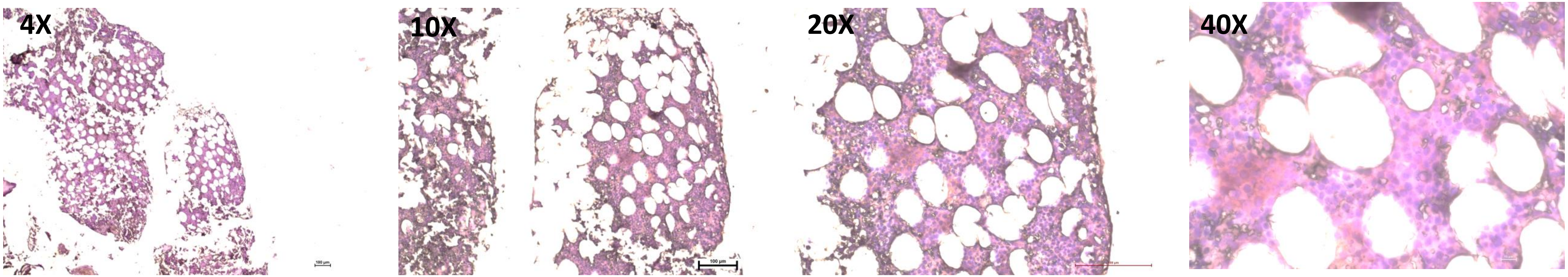

IPSS-R Low

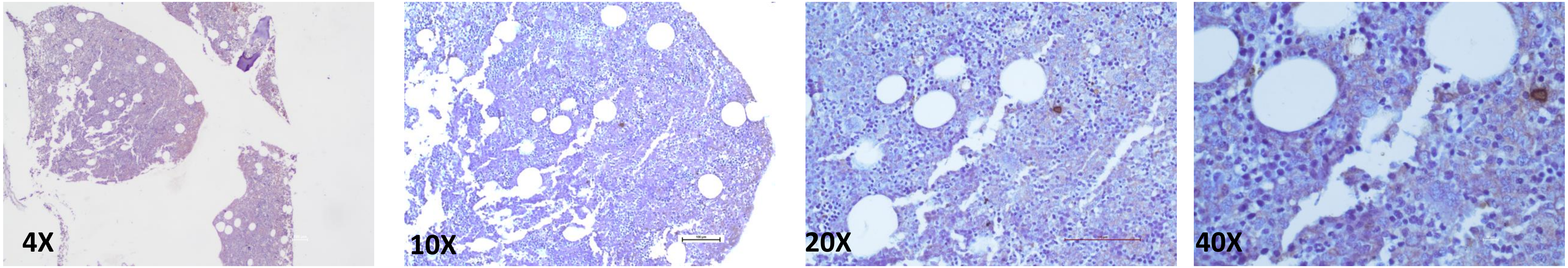

IPSS-R Low

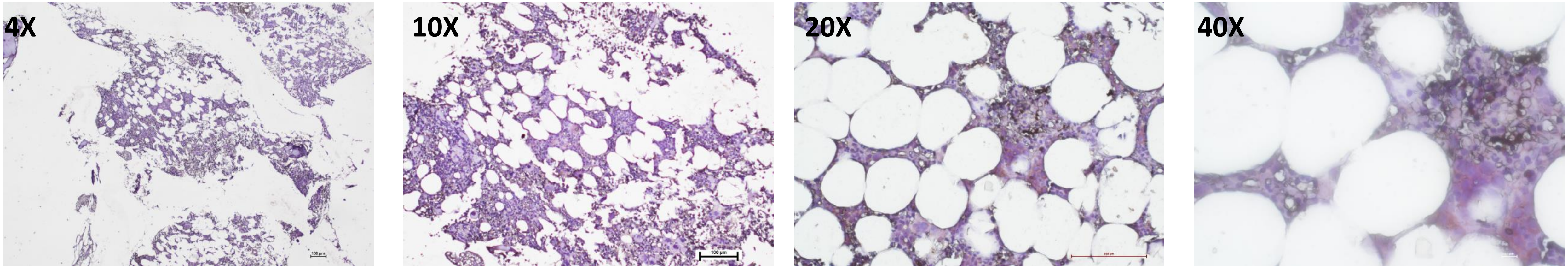

IPSS-R Intermediate

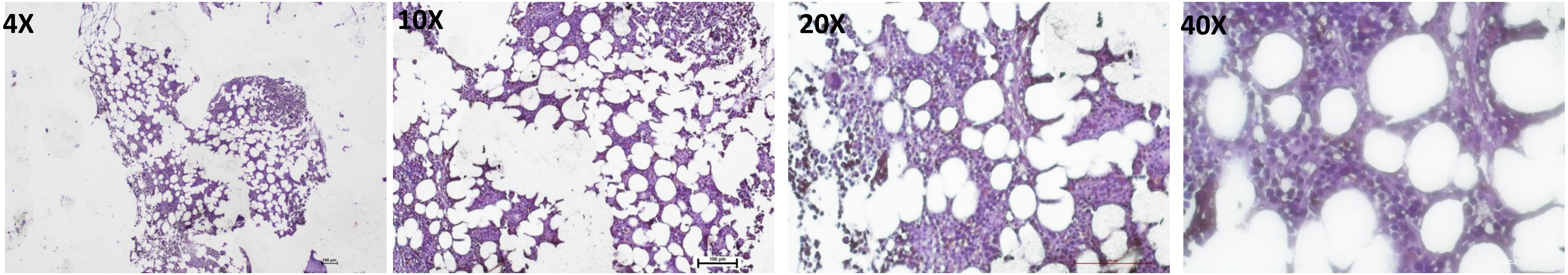

IPSS-R Intermediate

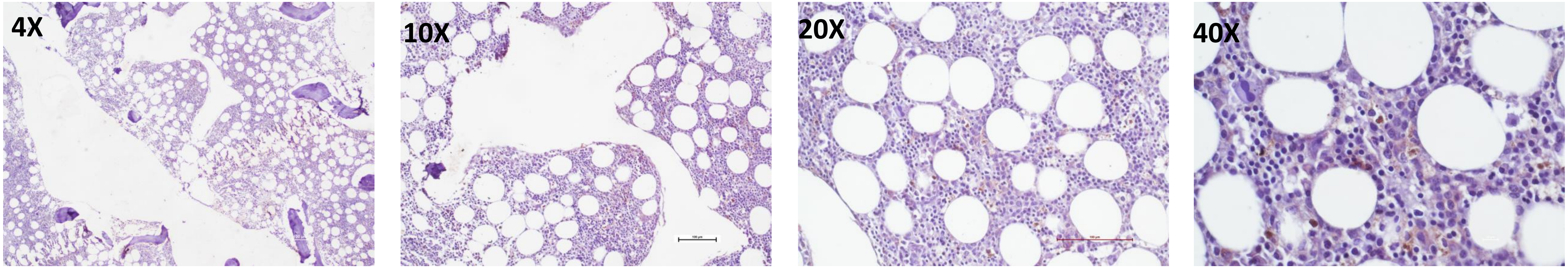

IPSS-R Intermediate

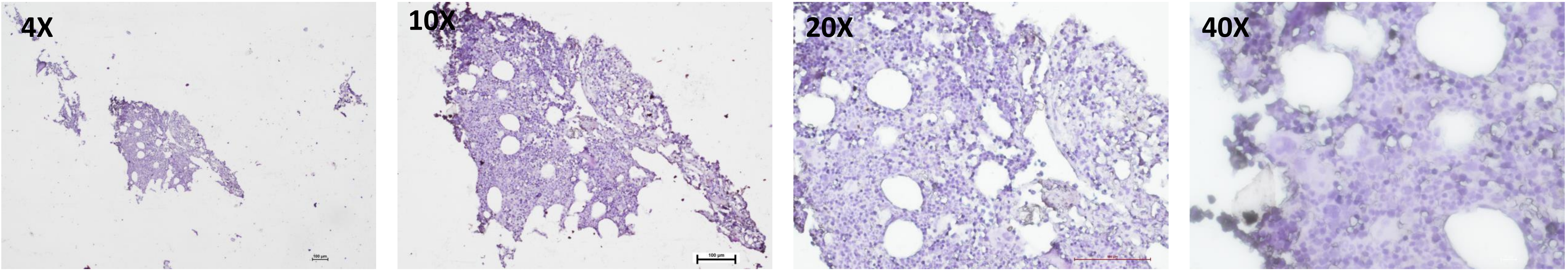

IPSS-R Intermediate

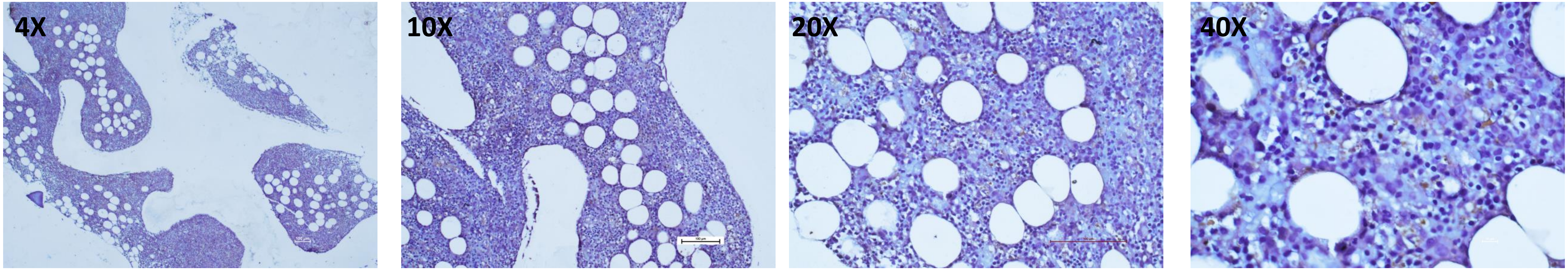

IPSS-R Intermediate

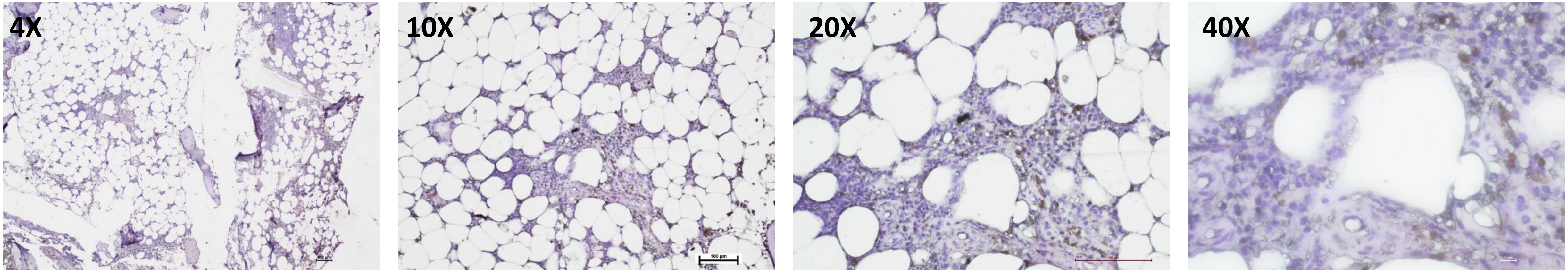

IPSS-R High

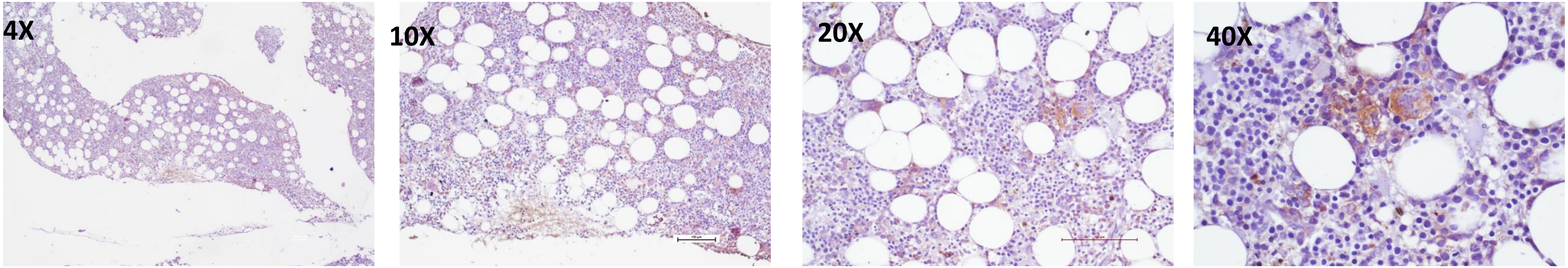

IPSS-R Very High

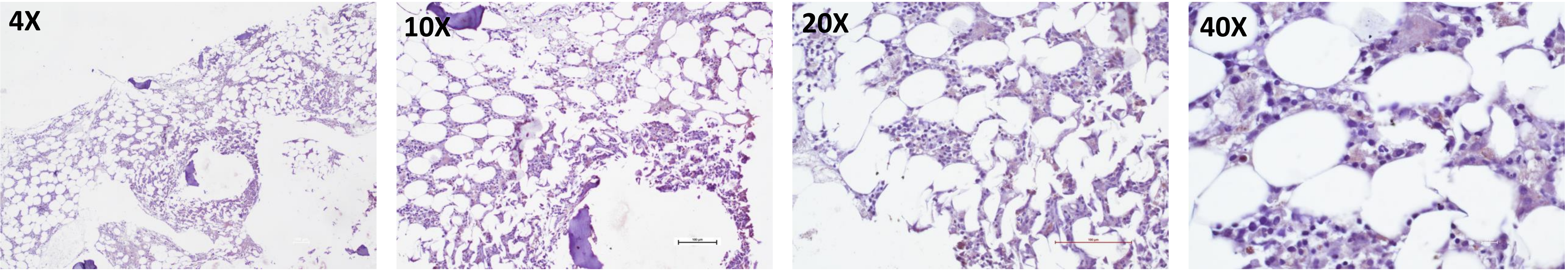

AML-MRC

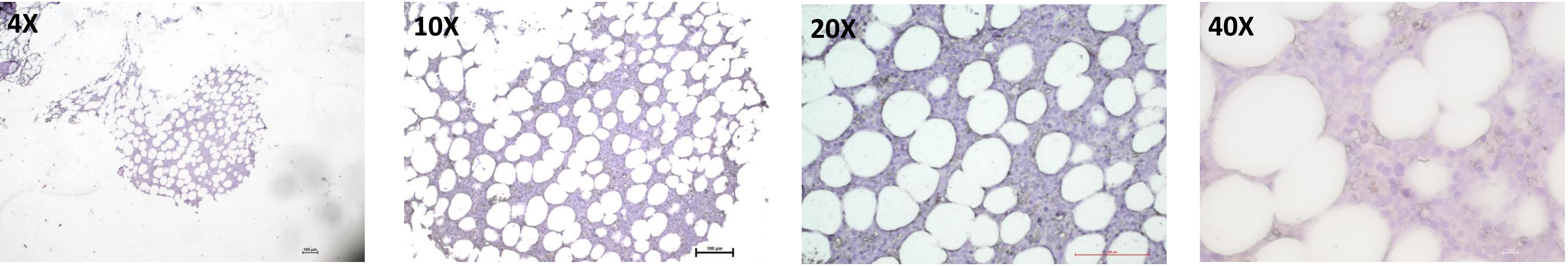

AML-MRC

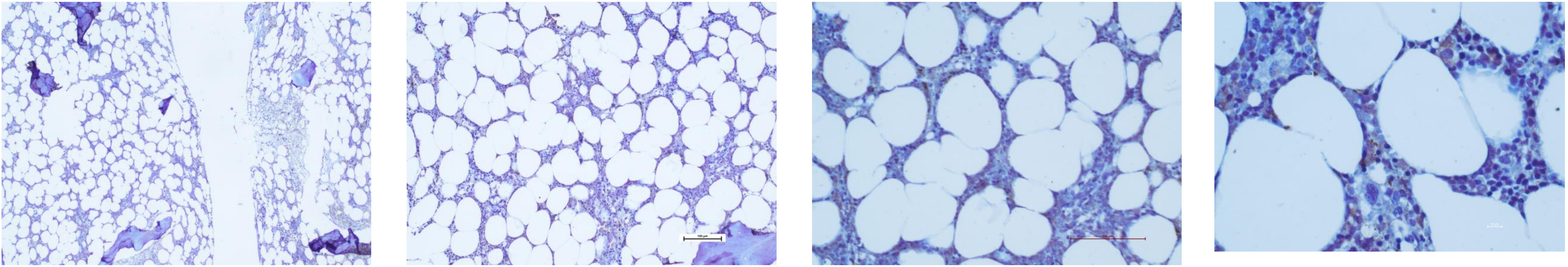

Supplementary Tables:

Alterations of the expression of *TET2* and DNA 5-hmC predict poor prognosis in Myelodysplastic Neoplasms: An observational study from India

Supplementary Table 1: List of primers used in this study

Gene	Primer	Annealing Temperature	Purpose
TET1	F: ACCTGCAGCTGTCTTGATCG	60°C	qPCR
	R: CACCCATGAGAGCTTTTCCCT		
TET2	F: AGGGACTGAGCTGCTGAATTC	60°C	qPCR
	R: GGGCTTCCATTCTGGAGCTT		
GAPDH	F: ACGGATTTGGTCGTATTGGG	60°C	qPCR
	R: CGCTCCTGGAAGATGGTGAT		
TET2-Exon 11	F: AATGGAAACCTATCAGTGGACAAC	60°C	PCR for Sanger sequencing
	R: TATATATCTGTTGTAAGGCCCTGTGA		
TET2-Exon 11	F: AATGGAAACCTATCAGTGGACAAC	60°C	Sanger sequencing
TET2-Exon 11	R: TATATATCTGTTGTAAGGCCCTGTGA	60°C	Sanger sequencing

Supplementary Table 2: Pathogenic variants of TET2 in patient samples

**Sample**	**Start: chr4**	**Ref**	**Alt**	**Type**	**Change: NM_001127208:**
**A2**	105234042	C	T	SNV	exon3:c.C100T:p.L34F
	105275570	AG	-	frameshift del	exon11:c.5060_5061del:p.Q1687fsX3
	105275843	A	G	SNV	exon11:c.A5333G:p.H1778R
	105276132	GT	-	frameshift del	exon11:c.5622_5623del:p.E1874fsX2
**A3**	105235006	G	A	SNV	exon3:c.G1064A:p.G355D
**V2**	105234028	C	G	SNV	exon3:c.C86G:p.P29R
**H3**	105276348	-	A	frameshift ins	exon11:c.5839dupA:p.G1946fsX2
**I1**	105275843	A	G	SNV	exon11:c.A5333G:p.H1778R
**I3**	105234028	C	G	SNV	exon3:c.C86G:p.P29R
**L1**	105234028	C	G	SNV	exon3:c.C86G:p.P29R

Continued…

**Sample**	**AA Change**	**ExAC_ All**	**1000g_ All**	**gnomAD_All**		**SIFT**	**Polyphen2 _HDIV**	**CADD_ phred**
				**Exome**	**Genome**			
**A2**	p.L34F	0.02	0.01	0.02	0.01	D	B	23
	p.Q1687fsX3	.	.	.	.	.	.	.
	p.H1778R	0.05	0.08	.	.	D	D	23.5
	p.E1874fsX2	.	.	.	.	.	.	.
**A3**	p.G355D	0.03	0.02	0.03	0.03	D	P	24.9
**V2**	p.P29R	0.06	0.09	0.06	0.04	T	D	24.2
**H3**	p.G1946fsX3	.	.	.	.	.	.	.
**I1**	p.H1778R	0.05	0.08	.	.	D	D	23.5
**I3**	p.P29R	0.06	0.09	0.06	0.04	T	D	24.2
**L1**	p.P29R	0.06	0.09	.		T	D	24.2

Legend: A: AML-MR, V: IPSS-R very high, H: IPSS-R high, I: IPSS-R intermediate, L: IPSS-R low
